# Pennsylvania Points the Way

**Published:** 1895-06

**Authors:** 


Pennsylvania Points the Way.—By the adoption of the act establishing a State Board of Veterinary Medical Examiners, the State of Pennsylvania gives notice to all future graduates that may desire to practise veterinary science within her borders that they, one and all, must come from Colleges maintaining at least a three-years' course of six months each, each session to be in different years. This equipment grants them the right to present themselves before the board for examination, and then will come the test as to how well this work has been done by those who compose the teaching-staff of the various colleges. This step by the Keystone State will surely be followed by many other States, under the new legislation or through amendments to existing laws, and dates the sure and early extermination of the two-year schools. How much better it would have been for all the schools to have kept just a little in advance of possible laws like this, for they have not come too soon; the country is filled to-day with an over-supply of half-equipped veterinarians, and the ignorance of the general public to this fact will deter for many months a refilling of the benches of the colleges until they better understand how narrow is the actual equipment.

